# Task-based functional MRI challenges in clinical neuroscience: Choice of the best head motion correction approach in multiple sclerosis

**DOI:** 10.3389/fnins.2022.1017211

**Published:** 2022-12-07

**Authors:** Júlia F. Soares, Rodolfo Abreu, Ana Cláudia Lima, Lívia Sousa, Sónia Batista, Miguel Castelo-Branco, João Valente Duarte

**Affiliations:** ^1^Coimbra Institute for Biomedical Imaging and Translational Research, Institute for Nuclear Sciences Applied to Health, University of Coimbra, Coimbra, Portugal; ^2^Neurology Department, Centro Hospitalar e Universitário de Coimbra, Coimbra, Portugal; ^3^Faculty of Medicine, University of Coimbra, Coimbra, Portugal

**Keywords:** head motion, correction of motion effects, task-fMRI, multiple sclerosis, neuroimaging

## Abstract

**Introduction:**

Functional MRI (fMRI) is commonly used for understanding brain organization and connectivity abnormalities in neurological conditions, and in particular in multiple sclerosis (MS). However, head motion degrades fMRI data quality and influences all image-derived metrics. Persistent controversies regarding the best correction strategy motivates a systematic comparison, including methods such as scrubbing and volume interpolation, to find optimal correction models, particularly in studies with clinical populations prone to characterize by high motion. Moreover, strategies for correction of motion effects gain more relevance in task-based designs, which are less explored compared to resting-state, have usually lower sample sizes, and may have a crucial role in describing the functioning of the brain and highlighting specific connectivity changes.

**Methods:**

We acquired fMRI data from 17 early MS patients and 14 matched healthy controls (HC) during performance of a visual task, characterized motion in both groups, and quantitatively compared the most used and easy to implement methods for correction of motion effects. We compared task-activation metrics obtained from: (i) models containing 6 or 24 motion parameters (MPs) as nuisance regressors; (ii) models containing nuisance regressors for 6 or 24 MPs and motion outliers (scrubbing) detected with Framewise Displacement or Derivative or root mean square VARiance over voxelS; and (iii) models with 6 or 24 MPs and motion outliers corrected through volume interpolation. To our knowledge, volume interpolation has not been systematically compared with scrubbing, nor investigated in task fMRI clinical studies in MS.

**Results:**

No differences in motion were found between groups, suggesting that recently diagnosed MS patients may not present problematic motion. In general, models with 6 MPs perform better than models with 24 MPs, suggesting the 6 MPs as the best trade-off between correction of motion effects and preservation of valuable information. Parsimonious models with 6 MPs and volume interpolation were the best combination for correcting motion in both groups, surpassing the scrubbing methods. A joint analysis regardless of the group further highlighted the value of volume interpolation.

**Discussion:**

Volume interpolation of motion outliers is an easy to implement technique, which may be an alternative to other methods and may improve the accuracy of fMRI analyses, crucially in clinical studies in MS and other neurological populations.

## Introduction

Resting-state functional MRI (rs-fMRI) has evolved to become one of the most common brain imaging modalities and has been crucial for understanding fundamental properties of brain organization and connectivity abnormalities associated with diverse clinical conditions (Filippi et al., 2019). Particularly, multiple sclerosis (MS) is a disconnection disease that is due to structural damage but also functional connectivity alterations, which has been extensively investigated with fMRI during rest (Sbardella et al., 2015; Shu et al., 2016; Eijlers et al., 2019; Meijer et al., 2020). However, task-designs target brain regions and networks that show distinct properties than in resting-state (Di et al., 2013; Schoonheim et al., 2015). Thus, task-fMRI may have a key role in describing the functioning of the brain, in highlighting specific connectivity changes, and thus in understanding this disease better. However, the blood oxygen-level-dependent (BOLD) signal measured with fMRI is highly susceptible to various sources of noise, such as head motion.

Motion artifacts degrade data quality and influence all image-derived metrics such as task activation and connectivity estimates (Zeng et al., 2014; Liu, 2016). On the one hand, rs-fMRI studies have demonstrated that head motion can introduce systematic bias to connectivity estimates by creating spurious but spatially structured patterns in functional connectivity (Power et al., 2014; Parkes et al., 2018; Maknojia et al., 2019). On the other hand, in task paradigms, which yield higher frequencies of brain signal changes that are closer to motion artifacts, head motion can be more challenging to deal with. For instance, when motion correlates/synchronizes with the experimental tasks it leads to false brain activations or a lower signal-to-noise ratio that can make it harder to detect a true activation of interest. If not properly accounted for, head motion will bias the statistical results, reducing the sensitivity and specificity for detecting task-specific BOLD responses (Seto et al., 2001; Power et al., 2014; Caballero-Gaudes and Reynolds, 2017).

To obtain a “clean” signal with neuronal and biological validity is then important to mitigate the effects of head motion. This is crucial in studies with developmental or clinical populations, especially those that tend to move more, where diagnosis and monitoring need to be the most accurate as possible (Griffanti et al., 2016; Saccà et al., 2021). Previous studies have shown that group differences in head motion between control and patient groups cause group differences in the resting-state network with rs-fMRI (Song et al., 2012; Lee et al., 2014; Maknojia et al., 2019; Saccà et al., 2019). Therefore, the presence of a neurological disease influences head motion and the optimal approach for correction of motion effects should be investigated in each specific context. In the particular context of MS, it has been reported that early diagnosed MS patients and patients with higher disability levels tend to move to a greater extent in the MRI scanner than control subjects (Boonstra et al., 2017; Saccà et al., 2018, 2019). A task-based fMRI study has found a linear increase in motion as task difficulty increased that was larger among MS patients with lower cognitive ability (Wylie et al., 2014). Furthermore, activation in the sensory-motor cortex during performance of a complex bilateral finger tapping task was also found to be greater in control subjects compared to relatively healthy MS patients, as a consequence of head motion in MS (Lowe et al., 2006). However, the effects of head motion in task-fMRI studies of MS, especially in early stages where head motion can be less evident but still present, and considering other task designs, were not systematically explored.

There is a plethora of methods described in the literature to correct head motion effects, which can be due to gradual head shifts and sudden movements of the head known as motion outliers. There are methods directly correcting the images for motion artifacts, either prospectively (Zaitsev et al., 2017; Huang et al., 2018; Maziero et al., 2020) or retrospectively (Glover and Pauly, 1992; Graedel et al., 2017; Rettenmeier et al., 2022). However, these are technically complex. The most common approach to compensate for the effects of head shifts is at the signal modeling level, after realigning all fMRI volumes to a reference volume (Power et al., 2015). The position of the head in space is described at each volume relatively to the reference volume using rigid body transformations by 6 motion parameters (MPs): translational displacements along X, Y, and Z axes; and rotational displacements of pitch, yaw, and roll. Then, these 6 MPs can be included as nuisance regressors in a General Linear Model (GLM) analysis of the fMRI data to account for the variance of the BOLD signal explained by the head shifts. However, because residual BOLD variance associated with head shifts can still be present, additional MP-derived regressors have been suggested, namely the temporal derivatives of the MPs (Power et al., 2013) and the quadratic terms, resulting in a total set of 12 MPs and 24 MPs, respectively (Turner et al., 1996; Satterthwaite et al., 2013). Additionally, motion outliers are more problematic and generate the most critical BOLD signal changes (Power et al., 2013). These can be identified as spikes in the data time courses and cause large variations in image intensity. Such spikes are not accurately estimated using rigid body transformations, and thus the realignment step or the regression of the MPs fails to account for them. As a solution, several metrics have been proposed for describing subject motion and the detection of motion outliers, the most common being the Root Mean Squared head position change (RMS movement), the Framewise Displacement (FD), and the Derivative or root mean square VARiance over voxelS (DVARS), with the latter being a particular form of the RMS. Power et al. (2013) compared the FD and DVARS metrics in terms of movement characterization and found that these provide very similar results, however, it was unclear whether one index captures data quality better than the other. In any case, when these summary statistics are above a certain threshold for a particular volume (e.g., values of 0.5 for FD and 0.5% ΔBOLD for DVARS), this volume is considered essentially unusable. Nonetheless, motion outliers can still be corrected through different ways, with the most common being censoring and scrubbing. Censoring is simply removing the outlier volumes from the data, which might result in biased samples (Parkes et al., 2018). Scrubbing follows a model-driven strategy, whereby the volumes affected by extreme motion are identified and additional scan nulling regressors (with 1 s at the volumes where motion spikes are detected and 0 s elsewhere) are regressed out from the fMRI either directly in the GLM as covariates or nuisance regressors, or *via* multiple regression where the output residuals constitute the signal free of noise (Siegel et al., 2014). Alternatively, volumes associated with motion outliers can be interpolated based on non-corrupted volumes (Mazaika et al., 2009; Tierney et al., 2016; Caballero-Gaudes and Reynolds, 2017; Mckechanie et al., 2019; Rudas et al., 2020).

Other approaches including realignment/tissue-based regression with 24 MPs, principal component analysis (PCA) or independent component analysis (ICA) methods (aCompCor and ICA-AROMA, respectively), global signal regression, and censoring of motion-contaminated volumes as described in Mascali et al. (2021) were compared for task-based functional connectivity. In Ciric et al. (2017) the same denoising pipelines plus spike regression (de-spiking) and scrubbing have been compared in a resting state framework, suggesting that different strategies may be appropriate depending on the context. The same methods were evaluated with data from clinical populations in Parkes et al. (2018). Despite all the worthy efforts, there is still no consensus regarding the optimal number of MP-related regressors to consider for tackling head shifts, nor the most appropriate additional approach to mitigate motion outliers (Zaitsev et al., 2015). Also, the volume interpolation method was not addressed in these previous studies and comparisons of the same approach but with different motion detection metrics (e.g., FD vs. DVARS) were not reported.

These issues raise the importance of these processing steps in functional connectivity studies where one wants to study functionally connected networks in task-based fMRI, due to the stimulation or cognitive processing irrespective of head motion. Thus, it is crucial to investigate the interaction effect of the disease and experimental design with strategies for correction of motion effects, to provide robust measures that might help to understand the pathophysiology of the disease and also serve as a tool for disease assessment of progression, ideally in a real-world clinical scenario. Taking this into consideration, we aim to characterize head motion and compare the most used correction strategies in clinical context using fMRI data collected from early diagnosed MS patients and healthy control subjects, during the performance of one visual passive task and one (more demanding) visual perceptual decision-making task. We started by computing head motion metrics for the two groups to study if there were relevant differences between groups. Next, we compared the most used strategies to correct the effects of head motion and tested if the group has influence on the choice of the correction method. The strategies we compared are models with 6 and 24 MPs to deal with head gradual movements, and models with 6 or 24 MPs plus methods for tackling motion outliers to investigate if these can provide a better correction than models with only 6 and 24 MPs. We compared scrubbing methods with two different motion outliers’ detection metrics, FD and DVARS, and volume interpolation. The best approach was determined based on the quality of the data analyses, given by activation and variance-explained metrics.

## Materials and methods

### Participants

All participants gave written informed consent to participate in the study after a full verbal and written explanation of the study. The study was approved by the ethics committees of the Faculty of Medicine of the University of Coimbra (reference CE-047/2018) and of the Centro Hospitalar e Universitário de Coimbra (CHUC) (reference CHUC-048-19), and the study was carried in accordance with the Code of Ethics of the World Medical Association (Declaration of Helsinki) for experiments involving humans. Patients were recruited and clinically assessed at the Neurology Department of the and met the criteria for MS diagnosis according to McDonald Criteria (Thompson et al., 2018). This study included 17 patients recently diagnosed with Relapsing Remitting MS (RRMS) and 14 healthy control (HC) subjects. Patients also underwent neuropsychological evaluation with the Brief International Cognitive Assessment for MS (BICAMS) (Langdon et al., 2012). Demographic data are presented in Table 1.

**TABLE 1:** Demographic data of the participants.

Group	N	Age	Gender	Handedness	EDSS	Disease duration (months)	Phenotype
MS	17	32.18 ± 8.05	6F	11R	2.05 ± 0.52	27.71 ± 23.26	RRMS
HC	14	30.75 ± 8.61	4F	8R	–	–	–

“F” stands for female, “R” stands for right. EDSS, expanded disability status scale.

### Experimental protocol

**FIGURE 1:**
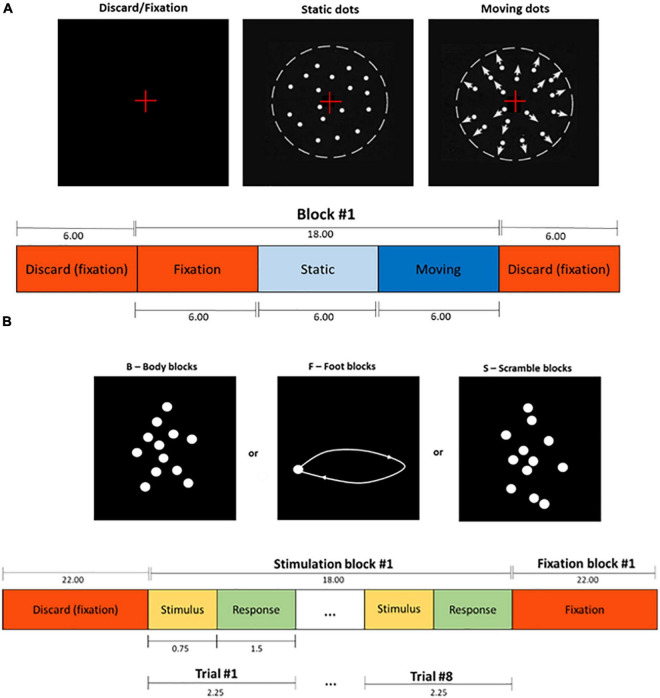
**(A)** Schematic representation of the functional localizer task. The duration of each period is indicated in seconds. Adapted from Huk and Heeger (2002). **(B)** Schematic representation of the biological motion (BM) task. The duration of each period is indicated in seconds.

The experimental protocol, illustrated in Figure 1, consisted of three functional runs: one run of a passive visual task, which was a functional localizer of the human middle temporal area [hMT+/V5, a low-level visual area well-known to respond to simple motion patterns (Peelen et al., 2006; Van Kemenade et al., 2014; Duarte et al., 2017)], and two runs of a decision-making visual task of biological motion (BM) perception (Duarte et al., 2022).

The localizer run consisted of 10 blocks of 18 s, with each block comprising three periods: the first was a fixation period with a red cross positioned at the center of the screen for 6 s. During the second period, a pattern of stationary white dots on a black background was shown for 6 s, followed by the third (and final) period during which the dots were moving towards and away from a central fixation cross at a constant speed (5 deg/sec) for 6 s.

Biological motion stimuli were built based on human motion capture data collected at 60 Hz, comprising 12 point-lights placed at the main joints of a male walker. Each BM perception run consisted of 12 blocks of 40 s: 4 or 5 blocks (depending on the starting block) of the point-light walker facing rightwards or leftwards (global biological motion), 4 or 5 blocks showing only the point-light located at the right ankle and moving rightwards of leftwards (local biological motion), and 3 blocks of point lights randomly positioned across the y axis, while maintaining their true trajectory across the x axis (scrambled motion). A total of 9 global, 9 local, and 6 random blocks were presented during the two BM perception runs. After each stimulus presentation, the participants reported the direction of motion of the dots (left or right) by pressing one of two buttons.

### Functional MRI data acquisition

Imaging was performed at the Portuguese Brain Imaging Network facilities (Coimbra, Portugal) on a 3T Siemens MAGNETOM Prisma Fit MRI scanner (Siemens, Erlangen, Germany) using a 64-channel RF receive coil. fMRI data were acquired using a 2D simultaneous multi-slice (SMS) gradient-echo echoplanar imaging (GE-EPI) sequence (6 × SMS and 2 × in-plane GRAPPA accelerations), with the following parameters: TR/TE = 1000/37 ms, voxel size = 2.0 × 2.0 × 2.0 mm^3^, 72 axial slices (whole-brain coverage), FOV = 200 × 200 mm^2^, FA = 68°, and phase encoding in the anterior-posterior direction. A short EPI acquisition (10 volumes) with reversed phase encoding direction (posterior-anterior) was also performed prior to each fMRI run, for image geometric distortion correction. A 3D anatomical T1-weighted MP2RAGE (TR = 5000 ms, TE = 3.11 ms; 192 interleaved slices with isotropic voxel size of 1 mm^3^) was also collected for subsequent image registration.

For each participant, 192 fMRI volumes were acquired during the functional localizer run, yielding 3.20 min of duration. The two BM runs comprised 507 volumes each, summing approximately 8.37 min in total.

### Functional MRI data preprocessing

Functional MRI data were preprocessed using custom scripts in MATLAB^®^, using the SPM12 software with CAT12 and PhysIO toolboxes (Kasper et al., 2017), and FMRIB Software Library (FSL). The preprocessing pipeline included: (1) slice timing correction; (2) realignment of all fMRI volumes relative to the first volume; (3) correction of geometric distortions caused by magnetic field inhomogeneity, with FSL tool TOPUP (Andersson et al., 2003); (4) bias field correction; (5) image registration (functional to structural); (6) segmentation of the T1 structural image (with CAT12 toolbox) to extract WM and ventricular CSF masks; (7) estimation of nuisance regressors (with PhysIO toolbox) such as cardiac and respiratory signals, WM and ventricular CSF average BOLD fluctuations and head motion (6 and 24 MPs and motion spikes); (8) Regression of noise fluctuations. Then the “clean images” from the regression were brain masked and the preprocessing was completed with spatial smoothing with a 3 mm full-width-at-half-maximum (FWHM) isotropic Gaussian kernel and high-pass temporal filtering with a cut-off period of 24 and 80 s for the localizer and BM tasks, respectively.

### Motion quantification

We characterized and compared head motion in both groups. For this characterization we individually computed the typical framewise displacement (FD), *meanFD*, the FD without considering the time series’ volumes affected by motion outliers, *meanFD’*, the FD considering only the time points where motion spikes were detected, *meanFD”*, the number of spikes, and the amount of variance of the average BOLD signal explained by motion, computed through the $R_{\text{adj}}^{2}\,\left(\frac{B\,O\,L\,D}{M\,o\,t\,i\,o\,n}\right)$ formula (see below).

Framewise displacement is a scalar quantity to express instantaneous head motion and it is computed through the time series of the 6 MPs obtained during the realignment step (Power et al., 2013). The FD is expressed by:


(1) $$\begin{array}{l} FD_{i}=\left|\text{Δ}d_{ix}\right|\;+\;\left|\text{Δ}d_{iy}\right|\;+\;|\text{Δ}d_{iz}|\;+\;\left|{\text{Δα}}_{i}\right|\\ \;+\;\left|{\text{Δβ}}_{i}\right|\;+\;\left|{\text{Δγ}}_{i}\right| \end{array}$$

where △*d*_*ix*_=*d*_(*i*−1)*x*_-*d*_*ix*_, and similarly for the other motion parameters, *d*_*ix*_,*d*_*iy*_,*d*_*iz*_,α_*ix*_,β_*ix*,_γ_*ix*_.

The FD was obtained with PhysIO toolbox. We computed the FD without considering the motion spikes and the FD considering only the spikes to understand how much the spikes would contribute to degradation of the BOLD signal due to intense movements. The number of spikes was given by the number of points detected by FD with motion above 0.5 mm.

Derivative or root mean square VARiance over voxelS is a measure computed from the BOLD signal itself and does not depend on the MPs. It represents how much the intensity of a volume changes in comparison to the previous one (Power et al., 2013). The DVARS metric is given by:


(2) $$D\,V\,A\,R\,S\,{\left(△\,I\right)}_{i}=\sqrt{\left\langle {\left[△\,I_{x}\,\left(\vec{x}\right)\right]}^{2}\right\rangle }=\sqrt{\langle [I_{i}\left(\vec{x)}-I_{i-1}{\left(\vec{x)}\right]}^{2}\right\rangle },$$

where $I_{i}(\vec{x)}$ is the image intensity at locus $(\vec{x)}$ on frame *i* and angle brackets denote the spatial average over the whole brain. The DVARS was computed with FSL tool *fsl_motion_outliers*, and motion outliers were identified by thresholding the DVARS at the 75th percentile plus 1.5 times the inter-quartile range.

We also computed the $R_{\text{adj}}^{2}\,\left(\frac{B\,O\,L\,D}{M\,o\,t\,i\,o\,n}\right)$measure as an additional metric to quantify motion between groups, which was estimated by the coefficient of determination adjusted for the degrees of freedom, defined according to Montgomery et al. (2012):


(3) $$R_{\text{adj}}^{2}\,\left(\frac{B\,O\,L\,D}{M\,o\,t\,i\,o\,n}\right)=1-\frac{N-1}{N-P-1}\,\frac{\sum_{i=1}^{N}\varepsilon_{i}^{2}}{\sum_{i=1}^{N}{\left(b_{i}-\bar{b}\right)}^{2}}$$

where $\bar{b}$ is the average BOLD signal, *N* is the number of volumes, and *P* the number of motion regressors; ε denotes the residual of the model under analysis, which is described by ε = b − β*X*, where β is the matrix containing the MPs, and β the associated weights estimated using a GLM framework. For each method (combination of MPs with scrubbing/interpolation) we computed the percentage of variation of the BOLD signal without correction for motion effects explained by the motion regressors. The higher the value of $R_{\text{adj}}^{2}$, the more variance of the BOLD signal is explained by motion, so the better is the method in capturing and correcting for head motion effects on the data.

Here, we tested 6 and 24 MPs because they represent the two extreme approaches complexity-wise (Maknojia et al., 2019). The 6 MPs were obtained during realignment and the 24 MPs which correspond to squares of the 6 MPs and temporal derivatives were obtained with PhysIO toolbox. Then we compared the different correction methods between groups based on quality metrics (described below). The goal is to identify which strategy, among the combination of 6 MPs or 24 MPs with scrubbing with FD, scrubbing with DVARS or volume interpolation is better to mitigate the effects of motion. Models with only 6 and 24 MPs were designed to understand which set of MPs is better for dealing with gradual movements. To correct the impact of gradual head motion, 6 MPs and 24 MPs are regressed out from the BOLD signal in the regression step of the preprocessing pipeline. The scrubbing method was implemented by identifying the motion spikes, through FD and DVARS with the thresholds mentioned above, with 1’s and 0’s elsewhere in the design matrix. Then these regressors are also regressed out from the BOLD signal in the regression step of the preprocessing pipeline. Volume interpolation was implemented with *ArtRepair* toolbox as the final step of the preprocessing where the affected volumes were firstly identified by FD with a threshold of 0.5 mm and then interpolated based on non-corrupted volumes. Finally, a signal free of motion-related noise is ready to be integrated in a General Linear Model (GLM) framework to obtain the statistical maps where the quality metrics will be computed to compare the different correction approaches.

### Statistical analysis

The GLM framework was used to map the regions involved in our tasks. It is basically a linear regression represented by:


(4) b=X⁢β+ε

with b the time series from one voxel, *X* the design matrix, β the model parameters, ε, the normally distributed error (or residuals) with zero mean (Pernet, 2014). Onsets and durations of each experimental condition were included in the model of the BOLD signal as regressors of interest representative of our tasks. For the localizer task we ended up with two regressors representing periods showing static points and moving points whereas for the BM tasks three regressors representing periods showing global biological motion, local biological motion, and scrambled motion were added to the model. These regressors were built based on unit boxcar functions with ones during the respective periods, and zeros elsewhere and convolved with a canonical, double gamma hemodynamic response function (HRF). The HRF-convolved regressors were then included in a GLM that was subsequently fitted to the fMRI data. After the fitting, the β weights are estimated, which represent the relevance of each regressor in explaining the variance of the data. Here, we set out to study brain regions that are activated when visual motion is present. Thus, the areas associated with these conditions were localized according to the contrasts [motion − static] and balanced [global BM motion + local BM motion + scrambled motion − baseline] for the localizer and BM runs, respectively. We used family wise error (FWE) correction for multiple comparisons based on Random Field Theory (RFT), and we only considered activations as significant those with a threshold of *p* < 0.05, with a cluster-level threshold of *p* < 0.05. One GLM was estimated for each participant and for each run, thus each participant ended up with 9 statistical maps per run: (i) map in which the only preprocessing step related to motion effects was realignment of the volumes to the first volume of the temporal series. These maps act as control to see how much motion-related noise was corrected with the different correction methods; (ii) map with 6 MPs; (iii) map with 24 MPs; (iv) map with 6 MPs and scrubbing with FD; (v) map with 6 MPs and scrubbing with DVARS; (vi) map with 6 MPs and volume interpolation; (vii) map with 24 MPs and scrubbing with FD; (viii) map with 24 MPs and scrubbing with DVARS; (ix) map with 24 MPs and volume interpolation. From the resulting activation maps, the quality metrics were extracted.

#### Quality metrics

The maximum (Z-max) and mean (Z-mean) Z-score values were extracted from each statistical map in each subject. Z-max is the highest Z-score value detected in the activation maps—region/cluster with the highest activation, Z-mean is the average Z-score value of all significant clusters in the activation map. These two measures are correlated but may not always follow the same tendency, i.e., a participant with a higher Z-max, when compared to others, may not have the higher Z-mean. The Z values indicate the sensitivity of the model in detecting brain regions that are associated with our tasks. The higher the values of Z, the higher is the accuracy of the correction method (Bosch, 2000). We decided to average the Z-score values of the three runs (one run of localizer task and two runs of BM task) because the Z-score values were very similar across the runs and the regions that activate in each one are the same, as expected, because participants performed visual motion tasks in both.

#### Comparisons

To statistically compare the amount of head motion between groups, a repeated measures design, a two-way ANOVA with one between subjects’ factor *Group* and one within subjects’ factor *Run* was applied separately to measures of FD, number of spikes, and $R_{\text{adj}}^{2}\,\left(\frac{B\,O\,L\,D}{M\,o\,t\,i\,o\,n}\right)\,\left(\mathrm{dependent}\,\mathrm{variables}\right)$. To evaluate the performance of the correction methods tested here, models of analysis of variance with repeated measures for the quality metrics, Z-max and Z-mean, were used. To compare the strategies mostly used to correct the gradual head shifts, a two-way mixed MANOVA (one between-subjects and one within-subjects factor) was performed. Similarly, to compare the strategies for correcting motion outliers’ effects and to study if scrubbing or volume interpolation methods are worth adding to the models with only 6 or 24 MPs for correction of gradual shifts, a three-way mixed MANOVA (one between-subjects and two within-subjects factors) was performed. The between-subjects factor in the two comparisons is *Group*, which has two nominal unrelated or independent categories: Multiple Sclerosis (MS) and control (HC) participants. For the first comparison, the within-subjects’ factor is the *MPs* (number of motion parameters), with two levels (6 MPs and 24 MPs) and for the second comparison, the within-subjects’ factors are the *MPs* and motion outliers’ *Correction Method*, with three levels (INTERP, FD, and DVARS). For both comparisons, the dependent variables are the quality metrics, Z-max and Z-mean. We included age as covariate for all statistical tests.

**FIGURE 2:**
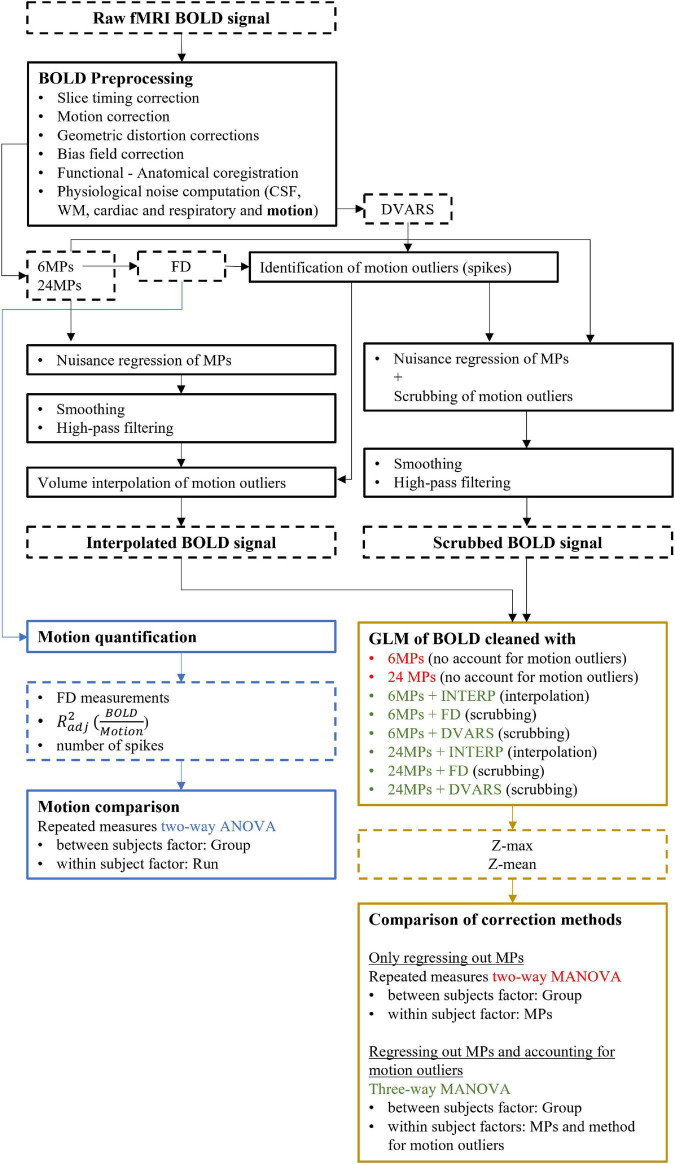
Workflow describing the methodology applied. Dashed outline and full outline boxes represent measures (outputs) and processes, respectively. Blue and yellow colors are related to motion quantification/characterization analysis and comparison of correction methods, respectively.

A workflow to facilitate the comprehension of the methodology applied is presented in Figure 2.

## Results

### Motion characterization

**FIGURE 3:**
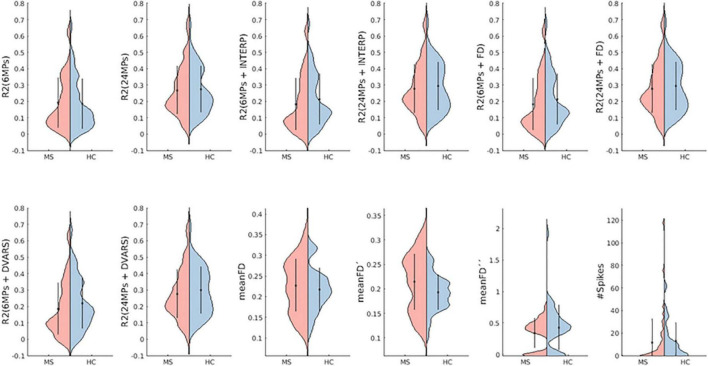
Motion quantification. Violin plots of motion metrics for both groups. *R*^2^ describes the amount of BOLD signal variation (without motion correction) explained by each set of regressors describing motion. Higher values of *R*^2^ means better performance of the method. meanFD is the mean framewise displacement, meanFD’ is the mean FD without considering motion outliers, meanFD” is the mean FD considering only the motion outliers. Higher values indicate more movement. #Spikes is the number of motion spikes. Red and blue represent the groups of MS patients and HC, respectively. The dots and vertical lines in each group represent the mean ± standard deviation of the values of the three runs. Both distributions are quite similar, evidencing no differences in motion metrics between groups, as supported by the ANOVA.

Motion characterization, evidencing FD measurements, $R_{\text{adj}}^{2}(\frac{B\,O\,L\,D}{M\,o\,t\,i\,o\,n}$) and the number of spikes for both groups, is represented in Figure 3. The two-way interaction for these metrics were not statistically significant: $p[R_{\text{adj}}^{2}(\frac{B\,O\,L\,D}{6\,M\,P\,s}$)] = 0.547; $p[R_{\text{adj}}^{2}(\frac{B\,O\,L\,D}{24\,M\,P\,s}$)] = 0.823; $p[R_{\text{adj}}^{2}(\frac{B\,O\,L\,D}{6\,M\,P\,s+I\,N\,T\,E\,R\,P}$)] 0.812; $p[R_{\text{adj}}^{2}(\frac{B\,O\,L\,D}{24\,M\,P\,s+I\,N\,T\,E\,R\,P}$)] = 0.82304; $p[R_{\text{adj}}^{2}(\frac{B\,O\,L\,D}{6\,M\,P\,s+F\,D}$)] = 0.812; $p[R_{\text{adj}}^{2}(\frac{B\,O\,L\,D}{24\,M\,P\,s+F\,D}$)] = 0.82304; $p[R_{\text{adj}}^{2}(\frac{B\,O\,L\,D}{6\,M\,P\,s+D\,V\,A\,R\,S}$)] = 0.768; $p[R_{\text{adj}}^{2}(\frac{B\,O\,L\,D}{24\,M\,P\,s+D\,V\,A\,R\,S}$)] = 0.768; *p* (meanFD) = 0.773; *p* (meanFD’) = 0.452; *p* (meanFD”) = 0.452; *p* (#Spikes) 0.764.

### Comparison of correction methods

The quality metrics of the models, group mean Z-max and Z-mean, for models with only MPs regressors for correction of gradual shifts are shown in Table 2. In Table 3 we present the metrics for the models with the combination of MPs for correction of gradual shifts and motion outliers’ correction methods.

**TABLE 2:** Metrics to assess the quality of the models using motion correction of gradual head shifts with 6 MPs and 24 MPs.

Correction	Group	Z-max	Z-mean
6 MPs	MS	8.59 ± 0.53	4.79 ± 0.49
	HC	8.34 ± 0.68	4.64 ± 0.38
24 MPs	MS	8.31 ± 0.62	4.64 ± 0.45
	HC	7.96 ± 0.89	4.49 ± 0.35

Values are presented as mean ± standard deviation in each group of participants.

**TABLE 3:** Metrics to assess the quality of the models using a combination of 6 MPs or 24 MPs with each method to correct the motion outliers’ effects.

			Metrics	
			
Correction	MPs	Group	Zmax	Zmean
INTERP	6 MPs	MS	8.61 ± 0.52	4.80 ± 0.49
		HC	8.36 ± 0.72	4.67 ± 0.38
	24 MPs	MS	8.31 ± 0.62	4.90 ± 1.08
		HC	7.96 ± 0.91	4.49 ± 0.34
FD	6 MPs	MS	8.53 ± 0.52	4.77 ± 0.51
		HC	8.28 ± 0.66	4.59 ± 0.36
	24 MPs	MS	8.25 ± 0.63	4.62 ± 0.46
		HC	7.87 ± 0.88	4.45 ± 0.34
DVARS	6 MPs	MS	8.55 ± 0.53	4.78 ± 0.49
		HC	8.24 ± 0.67	4.58 ± 0.38
	24 MPs	MS	8.26 ± 0.64	4.64 ± 0.46
		HC	7.81 ± 0.92	4.43 ± 0.33

Values are presented as mean ± standard deviation in each group of participants. “INTERP” stands for volume interpolation models, “FD” are the models with scrubbing using FD as the outliers’ detection metric, “DVARS” represents the models with scrubbing using DVARS as the outliers’ detection metric.

**FIGURE 4:**
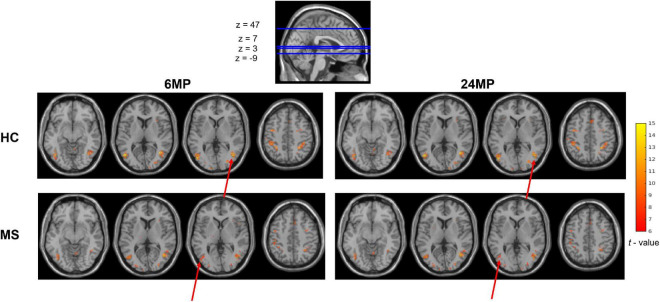
Group mean activation maps, resulting from the contrast [motion–no motion] in all runs. On the left are represented activation maps resulting from models with 6 MPs. On the right are represented the activation maps resulting from models with 24 MPs. Results are thresholded at a voxel *p*-value < 0.05, FWE corrected for multiple comparisons. The color bar scale represents *t*-values. We can observe slightly higher extent of significant activations (red arrow) in the maps with 6 MPs which is consistent with the results of the statistical analysis (higher *z*-values for 6 MPs models).

The two-way interaction of the two-way mixed MANOVA was non-significant *p* (Z-max) = 0.077; *p* (Z-mean) = 0.932. Subsequently, the main effect of *MPs* was significant (*p* < 0.001), with pairwise comparisons showing higher Z-scores for maps with 6 MPs, suggesting that using 6 MPs is better than using 24 MPs regardless of the group. Figure 4 shows mean activation maps of models containing 6 and 24 MPs for each group.

The three-way interaction from the three-way mixed MANOVA was non-significant, *p* (Z-max) = 0.535; *p* (Z-mean) = 0.052. The interaction *Method* and *Group* was significant, *p* (Z-max) = 0.045; *p* (Z-mean) = 0.435. As this interaction was significant, we computed simple main effects through a one-way MANOVA with one within subjects’ factor, *Method*, for each group. Pairwise comparisons showed the following order of outliers’ correction method performance for each group: in MS patients INTERP > DVARS > FD, *p* (Z-max) < 0.001 and *p* (Z-mean) < 0.001, although between DVARS and FD there are no significant differences, *p* (Z-max) = 0.281 and *p* (Z-mean) = 0.211; and in HC subjects INTERP > FD > DVARS, *p* (Z-max) < 0.001 and *p* (Z-mean) < 0.001, although between DVARS and FD only Z-max is marginally different, *p* (Z-max) = 0.045 and *p* (Z-mean) = 0.418. These results show that volume interpolation exhibits the best performance in both groups.

**FIGURE 5:**
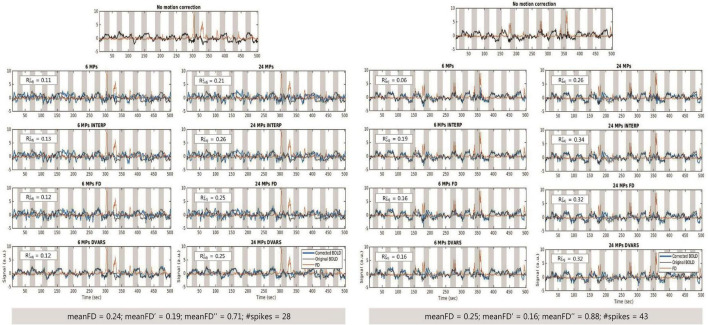
**(Left)** Time courses of the BOLD signal, before motion correction and after motion correction with each approach, and mean FD for one participant from the HC group. **(Right)** Time courses of the BOLD signal, before motion correction and after motion correction with each approach, and mean FD for one participant from the MS group. Measurements of FD allow the identification of spikes in the data, e.g., close to 300 s (HC participant) and 350 s (MS participant).

Figure 5 illustrates mean BOLD signal inside the Z-max cluster, located in the visual region hMT+, before and after correction of motion effects and mean FD time courses for one example participant of each group.

## Discussion

There is a lack of consensus regarding which is the best approach to mitigate the effects of head motion in task-fMRI data. Reaching a consensus on the best strategy is even more important in the clinical context to produce reliable interpretations and foster applications in neurology. In this study we compared different strategies to compensate for head motion in fMRI data in a group of MS patients and a group of HC performing two visual tasks. We found that the combination of 6 MPs with volume interpolation of motion outliers was the best correction approach in both groups.

### Characterization of head motion

We started by characterizing head motion in the two groups to study if the presence of disease affects motion occurrence. This comparison revealed that no significant differences in head motion were found between MS patients and HC, i.e., early diagnosed patients do not seem to move more than the HC participants. While previous studies found clear evidence of greater motion in patients with MS than in HC subjects (Wylie et al., 2014; Saccà et al., 2018), our results may be due to the fact that the participants in this study are in early stages of the disease, have lower levels of EDSS and therefore do not show significant physical disabilities. Furthermore, in this study patients with MS are cognitively preserved, while others have investigated patients with cognitive impairment and suggested that healthy individuals and cognitively preserved patients with MS may perform the cognitive task with enough efficiency that cerebral resources remain available for remaining still (Wylie et al., 2014). These authors have also shown a linear increase in movement of patients with MS and HC (to a less extent) as task difficulty increased. In this case, it might happen that our task is not demanding enough for these effects to stand out. Regarding the metrics we used to compare motion between groups, in addition to the well-known FD, we computed two variations of FD to investigate if the overall motion observed in each participant was mainly due to gradual head shifts or due the observation of motion outliers (abrupt motion spikes). The violin plots of these metrics show that values of FD without considering the points where motion outliers were detected are very similar to the conventional FD (all time points) values, supporting that in this cohort the abrupt movements of the head were not very problematic in overwhelmingly influence overall motion quantification. Nevertheless, it can be interesting and useful to check these motion metrics before deciding which correction approach should be put into practice.

### Comparison of strategies for correction of head motion effects

Next, we compared the most commonly used strategies to correct the effects of head motion. In this comparison we aimed to study: (1) if the group has influence on the performance of the correction method; (2) if including temporal derivatives of MPs would improve the correction of gradual movements; (3) if models with only these MPs regressors would be enough to compensate for all the head motion, even for the more abrupt movements; (4) if not, which combination, between number of MPs and scrubbing with FD, scrubbing with DVARS and volume interpolation would correct better the effects of motion outliers.

The comparison between correction approaches considering only 6 or 24 MPs revealed that higher Z-score values are obtained when considering 6 MPs regardless the group, which suggests that using 6 MPs is better than using 24 MPs. This recommends that task-specific brain regions are detected with higher sensitivity and less biased by noise due to a better correction when using 6 MPs relatively to using 24 MPs. The activation maps resultant from the GLM analysis show that maps with 6 MPs have slightly larger activations (more activated voxels) than maps with 24 MPs, even with the $R_{\text{adj}}^{2}$ being higher for the approaches with 24 MPs. The $R_{\text{adj}}^{2}$ indicates the amount of variance of the average BOLD signal without correction for motion effects that is explained by motion regressors. The higher the values, the more motion contributions are removed. However, too much variance might be removed with too many motion parameters, including information not related to motion, which seems to be the case as the approaches with 6 MPs have lower $R_{\text{adj}}^{2}$ values but higher Z-scores. This might sound counterintuitive, as adding more parameters to a model usually leads to overfitting. However, if the correlation between the 24 MPs is high, the result might be an “overcorrection.” Actually, these results are consistent with literature reporting that adding temporal derivatives can result in loss of degrees of freedom and therefore loss of valuable information (Power et al., 2015; Yang et al., 2019). In the context of this visual motion task, correcting head shifts with 6 MPs seems to be enough to cover the effects of gradual movements.

The traditional and common analyses rely only on a set of MPs as regressors to correct motion effects. However, to answer the question if models with only MPs regressors would be enough to compensate for all the head motion, even for the more abrupt movements, a third analysis was performed in which models with combinations of MPs and motion outliers’ correction methods were considered. The Z-score values are very similar between the models with only the MPs regressors and the rest of the models combining MPs with detection and correction of motion outliers. At a first glance this might mean that it’s not worth adding additional methods to compensate for the effects of more abrupt movements. However, actually because of that similarity and the fact that there is no considerable decrease in Z-values, which would suggest loss of valuable information, we consider that adding methods such as scrubbing, or volume interpolation might indeed crucial to eliminate residual noise and to compensate for putative motion outliers’ effects. The $R_{\text{adj}}^{2}$ values indicated in Figure 3 with an example of original and corrected time courses for one participant of each group also suggest that adding such methods help, indeed, to compensate for extra motion contributions without loss of signal of interest. Several authors have also reported the inadequacy of MP regressors to remove all motion artifacts, arguing that even expansions including terms from 3 time points (e.g., 36 MPs) leave much motion-related variance in data and therefore other more attractive methods are necessary to correct for motion effects (Power et al., 2014, 2015; Patriat et al., 2017).

This third analysis revealed that activation maps with higher Z-scores ended up being those that result from models with 6 MPs and volume interpolation in both groups. To our knowledge there are no previous studies with a direct comparison, in the same data, of scrubbing, which is a modeling strategy, and volume interpolation, although the two approaches are widely used. Tierney et al. (2016) have shown that cubic spline interpolation of motion corrupted volumes improves the quality of fMRI in healthy children that typically move in the scanner. In our study, we tested volume interpolation in a clinical context with a population prone to characterize for high motion. The fact that volume interpolation outperformed the scrubbing methods in both groups suggests that it is a robust method independently of the presence of disease and can be considered as an optimal method to improve future fMRI analyses. Thus, it is important to discuss the impact of modeling motion outliers and interpolation in the data. Modeling motion outliers through scrubbing is a widely used technique to correct sudden movements of the head, however, it creates temporal discontinuities as it involves some effective data loss, in which volumes to be regressed out do not contribute to the task-related parameter estimates, reducing the available degrees of freedom (Jones et al., 2021). Interpolation overcomes this problem and avoids side effects in the high pass filtering step (Michielsen et al., 2011). However, volume interpolation induces synthetic data, and the duration of the censored segment, as well as the type of interpolation (linear, Fourier, wavelets, or splines), may produce different effects that further depend on the choice of these parameters (Caballero-Gaudes and Reynolds, 2017). These effects and the negative impacts of using interpolation must be further investigated.

This analysis also allowed us to directly compare the performance between motion outliers’ detection metrics, FD and DVARS. Although the difference is not significant, FD appears to perform better in the HC group while DVARS is preferable in the MS group. Despite the easiness in producing either measure, it is presently unclear whether one index captures data quality better than the other (Power et al., 2013). Yet, this outcome might indicate that the best approach is dependent on the specific neurological disease, which reiterates the need of testing different correction methods in specific populations such as MS.

Considering our results, we suggest that the optimal method, which reflects the best compromise between homogeneity of methodology between groups and performance, is the combination of 6 MPs with outliers’ interpolation as it surpasses the performance of the common scrubbing methods. Moreover, this study represents a first step towards a more standard procedure for correction of head motion effects in fMRI studies in this context.

### Limitations

One limitation might be the fact that we used activation metrics to assess the quality of the correction approaches. However, we already knew *a priori* which regions would be involved in the processing of these tasks, since it is a network that has already been well-studied (Sokolov et al., 2018; Duarte et al., 2022) and the activation maps confirm the recruitment of such regions. Furthermore, to have a better ground truth, one would have to resort to simulated data or studies with more invasive methods, such as intracranial electroencephalography (iEEG), or combine techniques such as fMRI-iEEG to detect with high precision (both spatial and temporal) the activation of regions during task-performance.

Other possible drawback may be that this evaluation was limited to a single dataset with a relatively limited sample size, thus, these results should be seen as suggestive regarding the recommendations for future studies. It will be important in future studies to increase the size of the cohorts, namely by including participants with higher amplitudes of motion to see if these results hold and are generalizable Yet, to our knowledge there are no fMRI studies with focus on neurological disorders such as MS to identify the best approach for correcting head motion. So, this work is a first and crucial step towards this goal, especially because motion may be more present in this population, and it may benefit more of correction methods. Nonetheless, we recognize that MS can cause alterations in brain activity, which could bias the results. Also, other pathologies with different pathophysiology could lead to different results showing a different correction method as optimal. For instance, Parkes et al. (2018) evaluate different strategies to correct motion effects in independent samples of people with schizophrenia and obsessive-compulsive disorder and recommend volume censoring as the method that performs best. However, Parkes and colleagues did not compare volume interpolation with the other strategies. Thus, validation of these results is needed in future studies, namely in other healthy/patient cohorts alone with more data and considering other task designs.

Apart from more traditional ways to deal with head motion, there are other techniques that can be implemented. External optical tracking systems that constantly measure the position of the head or the use of dedicated sequences with navigator echoes or active markers are examples. These methods act directly on the k-space domain and correct motion effects during data acquisition (Zaitsev et al., 2017; Huang et al., 2018; Maziero et al., 2020; Rettenmeier et al., 2022). However, it is important to consider the context in which these methods are applied. Here, we focused our study on commonly and easily applicable methods, since we wanted to employ them in a clinical context. Nonetheless, the potential improvement of modeling the BOLD response that can be achieved with a combination of prospective and retrospective image correction methods should be investigated in future studies.

## Conclusion

In this study we characterized head motion in patients with early MS and healthy controls and compared different techniques to tackle head motion in task-based fMRI data to reach a consensus on the best strategies to use. There were no differences between groups in motion quantification metrics, and data analysis of quality metrics have shown that using 6 MPs and volume interpolation is the best correction approach. Nevertheless, this work suggests that the presence of a neurological disease might influence the optimal approach, which should be investigated in each specific context. This study is the first to systematically investigate the best approach for correcting head motion in MS, through comparison of commonly used and easy to implement approaches to correct head motion effects such as motion regression, scrubbing, and volume interpolation. Also, it is the first time that volume interpolation was compared with other methods which ended up showing its clinical value since it improved the accuracy of fMRI analyses, which is crucial in clinical neuroscience studies with patient populations.

## Data availability statement

The raw data supporting the conclusions of this article will be made available by the authors, without undue reservation.

## Ethics statement

The studies involving human participants were reviewed and approved by the Ethics Committee of the Faculty of Medicine of the University of Coimbra and the Ethics Committee of the Centro Hospitalar e Universitário de Coimbra and the study was conducted in accordance with the 1964 Declaration of Helsinki and its later amendments. The patients/participants provided their written informed consent to participate in this study.

## Author contributions

JS: data acquisition, analysis, and interpretation and manuscript writing. RA: data acquisition, analysis, and interpretation. AL: clinical and neuropsychological evaluation of MS patients. LS: recruitment and clinical evaluation of MS patients and manuscript review. SB: recruitment, clinical, and neuropsychological evaluation of MS patients and manuscript review. MC-B: study design, data interpretation, and manuscript review. JD: study design, supervision, data interpretation, and manuscript writing and review. All authors contributed to the article and approved the submitted version.
