# Supplementary material for: Task-based functional MRI challenges in clinical neuroscience: Choice of the best head motion correction approach in multiple sclerosis
Source: Front Neurosci. 2022 Dec 7;16:1017211. doi: 10.3389/fnins.2022.1017211 (PMC9768441; doi:10.3389/fnins.2022.1017211)
Supplement: Supplementary file 1 [file Image_1.pdf]

Supplementary Material

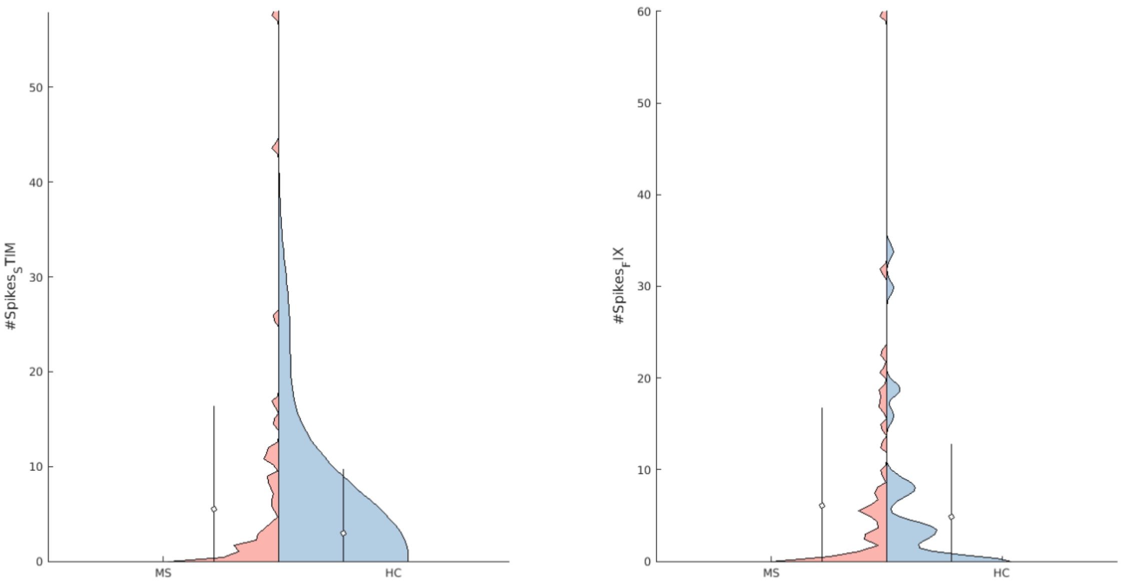

Supplementary Figure 1: (left) Violin plots of number of motion outliers during stimulation periods. (right) Violin plots of number of motion outliers during fixation periods. Red and blue represent the groups of MS patients and HC, respectively. The dots and vertical lines in each group represent the mean ± standard deviation. Both distributions are quite similar, evidencing no differences in number of motion outliers during stimulation or fixation periods between groups, as supported by ANOVA (*p*-value (#Spikes_STIM) = 0.14; *p*-value (#Spikes_FIX) = 0.16.
